# Carbon emission from Western Siberian inland waters

**DOI:** 10.1038/s41467-021-21054-1

**Published:** 2021-02-05

**Authors:** Jan Karlsson, Svetlana Serikova, Sergey N. Vorobyev, Gerard Rocher-Ros, Blaize Denfeld, Oleg S. Pokrovsky

**Affiliations:** 1 Climate Impacts Research Centre (CIRC), Department of Ecology and Environmental Science, Umeå University, Linnaeus väg 6, 901 87 Umeå, Sweden; 2 Swedish Geotechnical Institute, Olaus Magnus väg 35, 581 93 Linköping, Sweden; 3 BIO-GEO-CLIM Laboratory, Tomsk State University, Lenina 36, 634 050 Tomsk, Russia; 4 Swedish University of Agricultural Sciences, Lennart Hjelms väg 9, 75651 Uppsala, Sweden; 5 GET UMR 5563 CNRS, Geoscience and Environment, University of Toulouse, 14 Avenue Edouard Belin, 31400 Toulouse, France; 6 Institute of Ecological Problems of the North, N. Laverov Federal Center for Integrated Arctic Research, Russian Academy of Sciences, Nab. Severnoi Dviny 23, 163 000 Arkhangelsk, Russia

**Keywords:** Carbon cycle, Carbon cycle

## Abstract

High-latitude regions play a key role in the carbon (C) cycle and climate system. An important question is the degree of mobilization and atmospheric release of vast soil C stocks, partly stored in permafrost, with amplified warming of these regions. A fraction of this C is exported to inland waters and emitted to the atmosphere, yet these losses are poorly constrained and seldom accounted for in assessments of high-latitude C balances. This is particularly relevant for Western Siberia, with its extensive peatland C stocks, which can be strongly sensitive to the ongoing changes in climate. Here we quantify C emission from inland waters, including the Ob’ River (Arctic’s largest watershed), across all permafrost zones of Western Siberia. We show that the inland water C emission is high (0.08–0.10 Pg C yr^−1^) and of major significance in the regional C cycle, largely exceeding (7–9 times) C export to the Arctic Ocean and reaching nearly half (35–50%) of the region’s land C uptake. This important role of C emission from inland waters highlights the need for coupled land–water studies to understand the contemporary C cycle and its response to warming.

## Abstract (web summary)

Rivers and lakes are thought to be a major conduit of loss for the massive amounts of carbon locked away in high-latitude systems, but such losses are poorly constrained. Here the authors quantify carbon emissions from rivers and lakes across Western Siberia, finding that emissions are high and exceed carbon export to the Arctic Ocean.

## Introduction

Northern high-latitude regions are covered by numerous rivers^1^ and lakes^2^, together occupying up to ~16% of the land area^3^. At the same time, these regions store a significant amount of carbon (C) (~1672 Pg C), ~88% of which is stored in perennially frozen ground—permafrost^4,5^. At present, warming has accelerated in high-latitude regions with the mean annual temperature rising twice as fast as the global average^4,6^, making previously frozen permafrost vulnerable to thaw^3,7^. When permafrost thaws, it exposes substantial quantities of organic C, resulting in C degradation and atmospheric release of carbon dioxide (CO_2_) and methane (CH_4_)^6^. Large quantities of terrestrial inorganic and organic C are also exported to inland waters^8,9^, leading to additional CO_2_ and CH_4_ emission from the water surface into the atmosphere. The C emission from inland waters is a substantial component of the global C cycle^10–13^, yet for high-latitude regions such assessments are scarce^14,15^ and generally restricted to small catchments^16,17^, implying major uncertainties in the understanding of the high-latitude C cycle and its feedback on the climate system^18^.

**Fig. 1:**
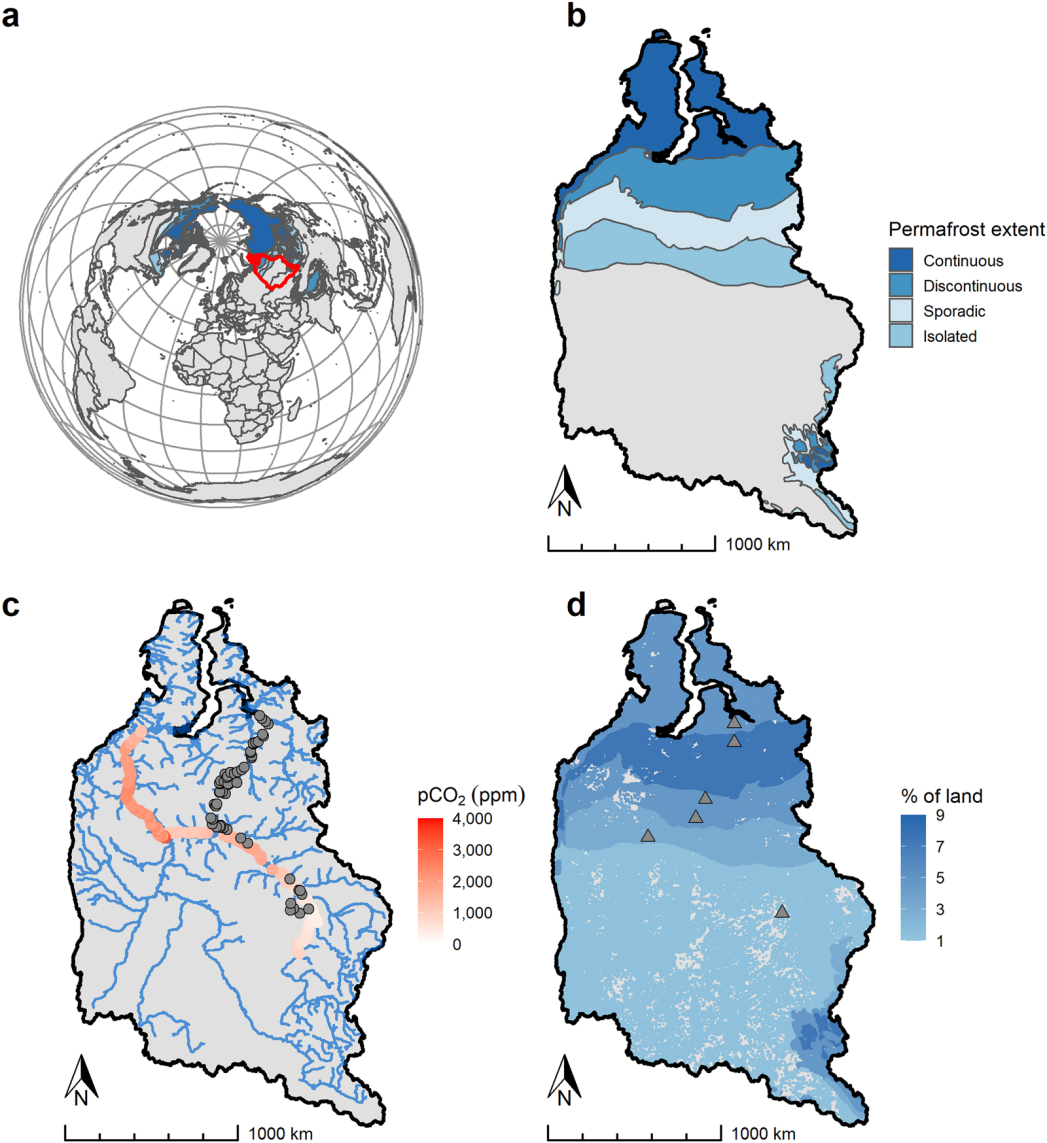
Characteristics of the study area. Map of Western Siberia with **a** location of the Ob’, Pur, and Taz River basins (red line), **b** permafrost extent^51^, **c** partial pressure of carbon dioxide (*p*CO_2_, ppm) in the Ob’ main channel (red gradient bar), distribution of main river network (blue lines)^1^ and location of 58 study rivers (gray circles), **d** lake abundance^2^ and location of 6 sites (gray triangles) with 89 study lakes.

Western Siberia with its extensive peatland area (~0.6 out of ~3.6 million km^2^)^19^ containing vast organic C stocks (~70 Pg C)^19,20^ partly underlain by permafrost (Fig. 1) is of particular interest in the high-latitude C cycle. Permafrost in Western Siberia is vulnerable to thaw and has been degrading over the last few decades^21^. Also, Western Siberia harbors the largest Arctic watershed, the Ob’ River, which is the second largest freshwater contributor to the Arctic Ocean^22^ and is one of the few Arctic rivers that traverse through all permafrost zones along its course (from permafrost-free to continuous permafrost zone). Importantly, no direct C emission estimate exists for inland waters of Western Siberia. Siberian systems have been included in global inland water C emission estimates^10^, but these are based on few indirect (calculated gas concentration and modeled fluxes) snapshot data with very low spatial and temporal resolution that have been found to introduce large uncertainties and cannot adequately capture annual C emissions^23–26^. Given the region’s large C stock and its overall sensitivity to warming, there is a clear need to estimate Western Siberian inland water C emission to constrain its role in present and future high-latitude and global C cycles.

Quantification of inland water C emission requires measurements of C emission rates and water surface areas of streams, rivers, ponds, and lakes—data that are scarce and geographically biased, thus limiting assessments of the role of inland water C emission at a regional scale. In this work we used new and recent estimates of inland water C emission rates (collected from 2014 to 2016) and areas across all permafrost zones of Western Siberia to quantify the total annual C emission from Western Siberian inland waters. Specifically, we used recently published C (CO_2_ + diffusive CH_4_) emission rates from 58 rivers^27^ and 89 ponds and lakes^28,29^ (hereby denoted “lakes” to distinguish from the extrapolated ponds, see below) covering a wide range of river (catchment area 2–150,000 km^2^) and lake (lake area 0.0001–1.2 km^2^) sizes and spanning a full gradient in permafrost extent (over 2000 km distance, Fig. 1 and Supplementary Table 1). We also included new estimates of C emission rates from the main channel of the Ob’ River based on the first-ever direct continuous measurements of partial pressure of CO_2_ (*p*CO_2_) (1546 ± 882 µatm, mean ± s.d., Fig. 1) and regional gas transfer coefficient (*k*) data. We obtained river and lake water surface areas in the Ob’ River basin and two other major Western Siberian rivers (Pur and Taz) from published databases^1,2^ and upscaled rivers and lakes C emission rates within each permafrost zone. Because the databases did not cover water surface areas of streams (lotic systems < 90 m wide) and the smallest ponds (lentic systems < 0.01 km^2^), we estimated these areas using Pareto law^1,30,31^ and upscaled streams and ponds C emission rates to the extrapolated areas. We summed C emission from all inland waters (rivers, streams, lakes, ponds) and assessed uncertainties using standard error propagation methods. The results allowed us to make the first assessment of the role of inland waters in the C cycle in one of the least studied but largest northern ecosystem in the world undergoing rapid permafrost thaw.

## Results and discussion

**Fig. 2:**
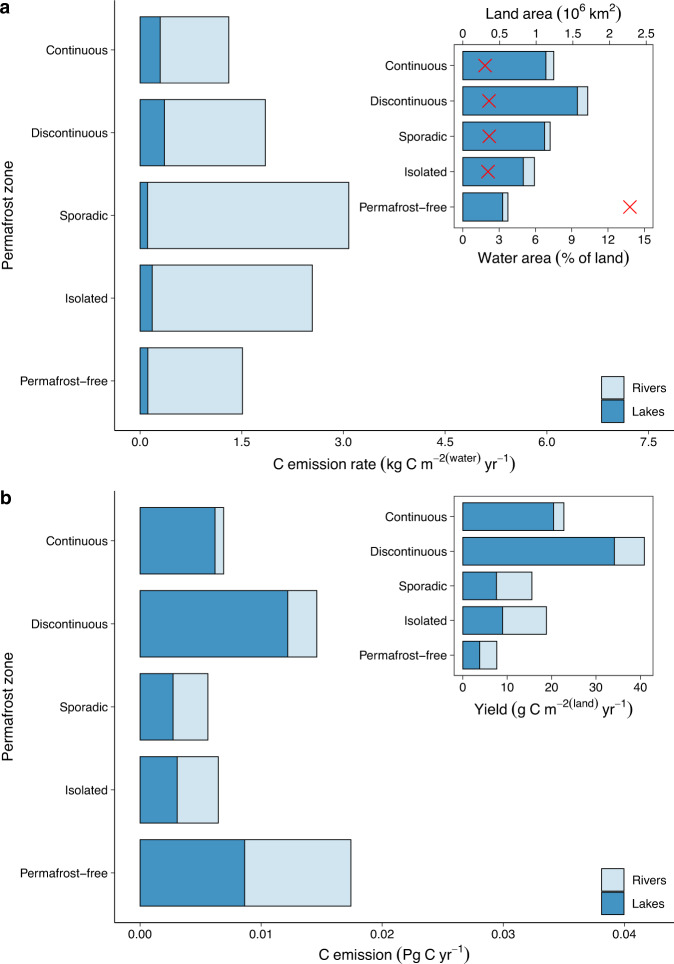
Inland water carbon (C) flux dynamics across permafrost gradient of Western Siberia. **a** Annual C emission rates per unit aquatic area and surface area coverage (inset) of rivers and lakes. Red crosses (inset) represent land area. **b** Annual C emission and yield (inset) from rivers and lakes. The figure does not include the smallest streams and ponds. For uncertainties see text, Table 1, and Supplementary Table 1.

We found that C emission rates from rivers are ~4-fold greater than C emission rates from lakes, resulting in greater yearly rates of C outgassing from lotic systems (rivers: 0.9 ± 0.5, lakes: 0.2 ± 0.1 kg C m^−2^ yr^−1^, mean ± s.d.) (Fig. 2). Taken together inland waters (excluding streams and ponds) cover ~5.2% of Western Siberia, with lakes (~171,000 km^2^) accounting for a major fraction of the landscape compared to rivers (~20,000 km^2^) (Table 1). The combined rivers and lakes C emission from Western Siberia was 0.050 (±0.007) Pg C yr^−1^ and showed not only high values across the entire region, but also differences among permafrost zones (Fig. 2) (*H* = 242.67, *P* < 0.05). The C yield (the total C emission from inland waters scaled to the land area) increased with increasing permafrost extent and reached its maximum in the discontinuous permafrost zone (Fig. 2) (*H* = 1556, *P* < 0.05). This pattern is a consequence of particularly high rates of C emission from lakes in the permafrost-rich zones combined with a large fraction of land area covered by lakes, while rivers contribute less to the C yield and exhibit no clear trend across permafrost zones. Such increase in C yield emphasizes the fact that, not only warm, but also cold permafrost-rich areas of Western Siberia are important contributors to the overall high inland water C emission from this region.

**Table 1:** Surface area and C emission (with associated uncertainties, see Methods) for inland waters of Western Siberia.

	Ob’ main channel	Rivers	Streams	Permafrost lakes	Permafrost-free lakes	Ponds
Area, km^2^	6831	12,919	13,639	96,089	74,940	254,956
C emission, Pg C yr^−1^	0.004 (±0.001)	0.013 (±0.003)	0.013 (±0.003)	0.024 (±0.006)	0.008 (±0.002)	0.039 (±0.010)

**Fig. 3:**
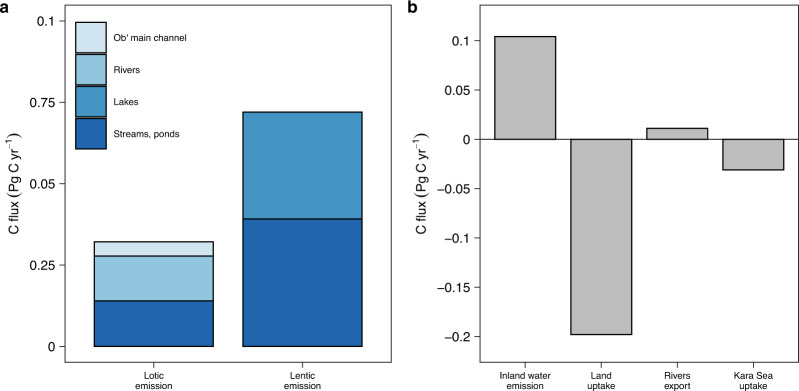
Carbon (C) fluxes of Western Siberia. **a** Different components of inland water C emission (blue shading). **b** Total inland water C emission, land C uptake, rivers C export to the Arctic ocean, and C uptake by the Kara sea. For uncertainties see text. Negative emission rates represent net uptake of CO_2_.

When assessing the magnitude of inland water C emission across Western Siberia we used published measurements of C emission rates and areas. However, these estimates do not include the areas of the smallest streams and ponds, which are commonly the most abundant water bodies in the landscape^2^ and potentially important sources of C to the atmosphere^16,32^. By extrapolating the areas of small systems^1,30,31^, the total lotic and lentic areas increased by ~1.6-fold (33,390 km^2^) and ~2.4-fold (425,986 km^2^), respectively, and increased the proportion of land occupied by water to ~12%. With the full size range of systems included, the C emission from inland waters increased ~2-fold, to 0.032 (±0.005) and 0.071 (±0.012) Pg C yr^−1^ for lotic and lentic systems, respectively (Fig. 3 and Table 1). The C emission from lotic systems was nearly equal between streams (0.013 ± 0.003 Pg C yr^−1^) and rivers (0.018 ± 0.003 Pg C yr^−1^), with C emission from the Ob’ main channel accounting for 24% (0.004 ± 0.001 Pg C yr^−1^) of river C emission. The C emission from lakes was slightly lower than from small ponds (0.032 ± 0.006 and 0.039 ± 0.010 Pg C yr^−1^, respectively) (Table 1). Taken together, the total C emission from Western Siberian inland waters amounted to 0.104 (±0.013) Pg C yr^−1^ (Fig. 3).

Our estimate for C emission from Western Siberian inland waters is greater than previously thought^10,33^. Specifically, mean *p*CO_2_ concentration, mean CO_2_ emission rate, and river C emission are ~3, ~6.3, and ~4.6-fold greater, respectively, than earlier assessment inferred from indirect observations and modeling^10,33^. Also, our estimate for total C emission from Western Siberian inland waters is ~1.4-fold greater than total C emission for this region and is ~2.6-fold greater than total C emission from other major Russian permafrost-draining rivers (i.e., sum of Kolyma, Lena, and Yenisei Rivers, 0.04 Pg C yr^−1^)^10^ derived based on modeling. Likewise, total C emission from Western Siberian inland waters is ~4.2-fold greater than total inland water C emission from the permafrost-affected Yukon River (0.02 Pg C yr^−1^) derived based on field observations^14^. These comparisons emphasize not only the fact that C emission from Western Siberian inland waters is high, but also highlight the need for additional regional estimates of inland water C emission from other major watersheds to better constrain their role in the global C cycle.

The results of this study are based on an extensive dataset with direct measurements of C emission rates covering a wide range of river and lake sizes and spanning over a complete permafrost gradient of Western Siberia. Yet, our estimate of total C emission from Western Siberian inland waters contains uncertainties related to practical constraints in carrying out measurements of C outgassing rates at higher spatial and temporal resolution. Measurements focused on key periods (spring, summer, autumn) likely captured main variability in C emission rates^24,34^, but still, collection of more temporally resolved data, especially in smaller systems, is needed to optimize sampling protocols for future C outgassing estimates. Furthermore, although the relatively homogenous geomorphology, soil type, and lithology of Western Siberia^35^ improve the likelihood of realistic extrapolation compared to more heterogeneous regions, additional spatially distributed data on C fluxes are needed, particularly in the permafrost-free zone (Fig. 1). Furthermore, due to the region’s flat terrain, Western Siberia has extensive floodplains and wetlands that increase substantially in water area during spring flood^36,37^. Assuming a maximal 85%^36^ increase in area over a period of 30 days, the C emission from inland waters of Western Siberia rises by ~11%. Our estimates also include uncertainties because of the lack of direct observations of smallest stream and pond areal distribution across the landscape. In particular, it has been recently suggested that contrary to stream area, pond area distribution in the landscape does not follow Pareto law^31,38^. Given this fact, we also adopted an alternative approach where we quantified pond C emission based on pond area derived from satellite image analysis of several sites within specific permafrost zones of Western Siberia^31^. Our result obtained with this approach yielded a ~31% lower estimate of total C emission (0.076 Pg C yr^−1^) from Western Siberian inland waters. Although these data are only based on a fraction of the basin, it still suggest a critical knowledge gap and the need for more detailed satellite inventories of pond area distribution across the landscape to assess their role in total inland water C emission.

This study also highlights the complexity in assessments of contemporary and future C outgassing from inland waters. First, it stresses the need to account for variability in both C emission rates and surface areas of streams, rivers, ponds, and lakes across the landscape. Second, the observed patterns in C emission and yield (Fig. 2) suggest that C outgassing is controlled differently between inland waters, yet detailed mechanistic studies are generally lacking. Current knowledge of these systems indicates a strong sensitivity on climate-dependent processes for C outgassing, but not always as expected based on knowledge from other regions. In general, C emission from rivers has been explained as mainly driven by lateral input of terrestrial C, and its subsequent mineralization and evasion from the water column^27^. These processes are largely controlled by temperature and water transit times that result in nonlinear dynamics with elevated C emission rates in warm vs cold permafrost regions, but also lower rates in permafrost-free zone^27^. For ponds and lakes lateral inputs of terrestrial C seem less important for C outgassing because of the generally small catchments. Instead, high rates of C emission from ponds and lakes have mainly been attributed to the shallow depths that cause relatively high mineralization of terrestrial organic C in their bottom sediments, with elevated rates in cold permafrost-rich regions because of high organic C quality of recently thawed sediments and hampered algae CO_2_ fixation^28^. Importantly, warming likely changes the areal coverage of inland waters, chiefly for thaw ponds and lakes where spatiotemporal variability in losses and gains create large uncertainty in the net outcome^3^. The complexity in the control of C outgassing from inland waters, with multiple drivers that vary across systems, implies that predicting future C outgassing from inland waters of Western Siberia is fraught with large uncertainties that require an interdisciplinary approach.

To estimate the relative importance of Western Siberian inland water C emission, we compared the total inland water C emission (0.076–0.104 Pg C yr^−1^) with other components of the regional C cycle (Fig. 3). First, we quantified Western Siberian land C uptake using regional data on terrestrial net ecosystem exchange (NEE) during 2016^39^ to −0.198 ± 0.009 Pg C yr^−1^ (Supplementary Fig. 1 and Supplementary Table 2). Thus, almost half (35–50%) of land C uptake is released back to the atmosphere via inland waters, implying that neglecting inland waters will largely overestimate the C sink strength of the region. Second, we compiled published data on river dissolved organic and inorganic C export to the Arctic Ocean (Ob’ River: mean for the period of 2003–2009; Pur and Taz Rivers: mean for the period of 2013–2014)^35,40–42^ (Supplementary Table 3) to 0.011 Pg C yr^−1^, i.e., 6.8–9.0-fold lower than C emission from inland waters. This implies that only ~10% of the C lost laterally from land reaches the Arctic Ocean, the rest is largely processed and emitted to the atmosphere by inland waters. Third, we found that the inland water C emission was ~2.4–3.0-times higher than the C uptake by the Kara Sea (−0.031 Pg C yr^−1^ during 2014)^43,44^ into where all Western Siberian rivers discharge. Because of interannual variability in fluxes, these types of comparisons should optimally include multiyear overlapping time periods, and thus the exact numbers should be treated with caution. Despite the uncertainties, these results emphasize the important role of C emissions from inland waters in the regional land–water C cycle. Ignoring C outgassing from inland waters may largely underestimate the impact of warming on these regions and overlook their weakening capacity to act as terrestrial C sinks. Although few coupled land–water C cycle studies exist for comparison, these data suggest that the role of inland waters of Ob in the C cycle are particularly high compared to other large scale estimates at high latitudes^14,45,46^ and globally^13,47^, and are on par with estimates for the Tropics^11,48^. The high significance of the inland waters of Western Siberia in the C cycle is likely a result of the overall flat terrain, which leads to relatively high water coverage and long water transit times, and thus favorable conditions for mineralization and outgassing of land derived C in inland waters^27,28^. Further studies on the coupled land–water C cycle are needed in order to improve the understanding of regional differences in the contemporary C cycle and predictions of future conditions in these understudied and climate-sensitive areas.

## Methods

### Inland water area estimates

We used available Global River Widths from Landsat (GRWL)^1^ as well as Global Water Bodies (GLOWABO)^2^ databases to estimate river and lake area in the Ob’, Pur, and Taz River basins. We first clipped the databases’ files to the respective area of Western Siberia using ArcMap 10.5. Then we overlaid GRWL river network with Ob’ main channel mask derived from World Major Rivers file (https://www.arcgis.com/home/item.html?id=44e8358cf83a4b43bc863646cd695945) by selecting features that are within ~20 km distance from the Ob’ main channel mask, and clipped Ob’ main channel from GRWL river network. We further separated the Ob’ main channel, river, and lake files to the respective permafrost zones using shapefiles of the Circum-Arctic Map of Permafrost and Ground Ice Conditions (http://nsidc.org/data/docs/fgdc/ggd318_map_circumarctic/). After that we merged the Ob’ main channel, river, and lake files for each permafrost zone in R (version 3.5), making three individual continuous spatial datasets with all permafrost zones present (Ob’ main channel spatial dataset consisting of 201,874 observations of river area, river spatial dataset of 882,124 observations of river area, and lake spatial dataset of 973,780 lakes). We excluded rivers <90 m wide from both river and Ob’ main channel datasets as done in Allen et al.^1^.

To include rivers and streams <90 m not present in the GRWL database, we estimated their surface area using Pareto extrapolation based on the specific Pareto shape parameter of 0.93 ± 0.0004 (±s.d.) reported for the rivers of Ob’ River basin^1^ and the minimum width of the first-order streams of 0.32 ± 0.077m ^49^. Similarly, to include ponds <0.01 km^2^ not present in the GLOWABO database we used Pareto extrapolation based on the specific Pareto shape parameter of 1.19 ± 0.0004 for the lakes of Ob’ River basin (obtained by fitting power law to Ob’ basin lakes in GLOWABO) and the smallest measured pond area of 0.000115 ± 0.0001 km^2^ (based on our field observations). There exist no data that enable to incorporate temporal variability in surface area across all systems and full region, and all areal estimates are assumed to represent average conditions over the open water season.

Since it has recently been suggested that contrary to stream area, pond area distribution in the landscape does not follow Pareto law^31,38^, we also quantified pond area using the fraction of land covered by these types of water bodies for each of the permafrost zones derived from lake satellite inventories of few sites within specific permafrost zones of the region from Muster et al. 2019^31^. Since Muster et al.^31^ estimated lake area (including ponds >0.0001 km^2^) in one site in the sporadic permafrost zone and in two sites in the continuous permafrost zone, we assumed the fraction of land covered by ponds in permafrost-free and isolated permafrost zones being similar to the sporadic permafrost zone (where ponds occupy ~2.9% of land), while the fraction of land covered by ponds in the discontinuous permafrost zone being similar to the average fraction of land covered by such water bodies between the two sites in the continuous permafrost zone (~0.62% of land).

### Ancillary data

We used published data on annual flow-weighted dissolved organic C (DOC)^35,40^ and dissolved inorganic C (DIC)^41,42^ export to the Arctic ocean during 2003–2009 for the Ob’ River, as well as annual flow-weighted DOC and DIC^41,42^ export (mean for the period of 2013–2014 quantified based on discharge data over the period 1971–1980) for Pur and Taz Rivers and summed them together to obtain the downstream C export for this region (Supplementary Table 3). We also estimated season length across entire region of Western Siberia by using a linear relationship between latitude (°N) and number of ice-free season days published for rivers^27^ and lakes^28^.

### C emission from Ob’ main channel

The *p*CO_2_ of Ob’ main channel was collected at 0.5 m depth every minute during 5 min at 10-min interval (yielding 4938 measurements) from a ship in summer 2016 (31 July to 11 August) by an infrared gas analyzer (Vaisala GMP222; accuracy ±1.5%) connected to a Campbell logger. The probe was calibrated in the lab and the *p*CO_2_ data were corrected for pressure and temperature (collected at same frequency) as described in Serikova et al.^27^. We estimated molar concentrations of *p*CO_2_ in water and in water in equilibrium with the atmosphere using Henry’s constant and pressure, and the average atmospheric concentration of 390 ppm. We grouped measurements and calculated the total CO_2_ evasion in each permafrost zone (permafrost-free, isolated, sporadic, discontinuous, and continuous). Since we lacked values in the continuous permafrost zone (because the ship finished sampling before reaching the Ob’ River mouth in the continuous permafrost zone), we used the values from the adjacent discontinuous permafrost zone. In total, we obtained 4396 *p*CO_2_ measurements in the Ob’ main channel, the number of measurements in each permafrost zone were 1516, 1982, 431, and 467 in the absent, isolated, sporadic, and discontinuous permafrost zones, respectively. To calculate the total CO_2_ evasion for each permafrost zone, we performed a Monte Carlo simulation by randomly sampling the observations of *p*CO_2_ in the water, *p*CO_2_ in the atmosphere and pH 100,000 times. The pH values for the respective permafrost zones were derived from our published river seasonal data^27^, truncating pH values to <8 because observed seasonal river pH values never exceeded a pH value of 8^27^. Thus, CO_2_ emissions were calculated as:

1 $${\mathrm{CO}}_2\;{\mathrm{emission}}\;{\mathrm{rate}} = \alpha \times {\mathrm{median}}\left( k \right) \times \left( {p{\mathrm{CO}}_{2\;\rm{water}} - p{\mathrm{CO}}_{2\;\rm{atmosphere}}} \right) \times 10^{ - 6}$$

where *α* is the pH-dependent chemical enhancement factor of CO_2_^50^, *k* is a median gas transfer coefficient of 4.464 m d^−1^ measured in four largest rivers (June 2015) of the Ob’, Pur, Pyakupur, and Taz Rivers (n = 39, each consisting of multiple measurements with floating chamber drifting in the middle of the river channel for 5 min), *p*CO_2 water_ is assigned *p*CO_2_ in the water and *p*CO_2 atmosphere_ is the assigned *p*CO_2_ in water in equilibrium with the atmosphere. To obtain the total yearly CO_2_ emissions in each permafrost zone, we multiplied the areal CO_2_ emission rate by the total river area in each permafrost zone, as well as the average ice-free season length based previous measurements within permafrost zones^27^. Furthermore, given our observations on seasonality in *p*CO_2_ concentrations in the Ob’ main channel from our previous work^27^ and considering that the ship *p*CO_2_ data sampling took place in July–August, we increased assigned *p*CO_2_ values (that represent summer *p*CO_2_) by a factor of 2 to get the approximate *p*CO_2_ in the spring, and took the average between them for quantifying daily rates of CO_2_ outgassing during open water period (May–October).

We estimated daily rate of CH_4_ outgassing for each of the points by using the median fraction of CH_4_ in C emission rate from our river data^27^ equal to 1.19% and summed up CO_2_ and CH_4_ emission rates to get the C (CO_2_ + CH_4_) emission rate (Supplementary Fig. 2). After that we multiplied C emission rate with respective season length and water areas for each of the data points, and summed all points together. We also added land area to different permafrost zones based on Circum-Arctic Map of Permafrost and Ground Ice Conditions^51^ and estimated C yield for each of the data points by normalizing the C emission rate to the respective land area.

### C emission from rivers and streams

To quantify C emission for rivers, we used published data on daily rates of CO_2_ outgassing from 58 rivers (*n* = 116)^27^. We created five normal distributions with CO_2_ emission rates for each permafrost zones with 10,000 values in each using the mean and s.d. for the respective permafrost zones from the observed CO_2_ emission rates river data (Supplementary Table 1). Then, for each observation of river area (*n* = 882,124), we randomly assigned a CO_2_ emission rate by subsampling the permafrost-specific distribution of emission rates defined above. After that we estimated daily rates of CH_4_ (i.e., 1.19% of total C emission) and C emission using the same approach as for Ob’ main channel. Then we estimated season length for each data point using the linear regression with latitude (*R*^2^ = 0.99, *F*_1,114_ = 7899.51, *p* < 0.01, Supplementary Table 4), and multiplied daily C emission rate with season length and water areas for each of the data points. Finally, we summed all points together to get the total C emission for rivers. We also added land area to different permafrost zones following the same approach as for the Ob’ main channel and estimated C yield. We quantified C emission for streams using the published^27^ median C emission rate of 5.67 g C m^−2^ d^−1^ for watersheds <100 km^2^ (assuming the same 1.19% fraction of CH_4_ in C emission rate). We then multiplied this value with extrapolated stream area and a median season length of 180.6 days was observed in our river data^27^.

### C emission from lakes and ponds

For permafrost-affected lakes we used published lake C (CO_2_ + diffusive CH_4_) emission rates data (76 lakes, *n* = 228, Supplementary Fig. 3)^28^. Since we did not observe linear dependence of C emission rates with lake size (*n* = 182, *R*^2^ = 0.00, *F*_1,179_ = 0.598, *p* > 0.05), we utilized a similar approach for upscaling C emission rates as in river upscaling. We created five normal distributions with C emission rates data representing different permafrost zones with 10,000 values in each using mean and s.d. for the respective permafrost zones from lake data (Supplementary Table 1)^28^. Then, for each observation (*n* = 612,003), we randomly assigned a CO_2_ emission rate by subsampling the permafrost-specific distribution of emission rates defined above. Then we estimated season length for each data point using the linear regression with latitude (*R*^2^ = 0.96, *F*_1226_ = 6012.09, *p* < 0.01, Supplementary Table 1), and multiplied daily C emission rate with season length and water areas for each of the data points. Finally, we summed all points together to get the total C emission for permafrost-affected lakes. We also estimated C yield following methods described above. When quantifying C emission for lakes in the permafrost-free zone, we used published C emission rates from 13 permafrost-free lakes (*n* = 13)^29^. Considering that the linear dependence of C (CO_2_ + diffusive CH_4_) emission rates on lake size in permafrost-free area was very weak (log_10_-transformed, *n* = 13, *R*^2^ = 0.20, *F*_1,11_ = 2.801, *p* > 0.05), we used the median C emission rate of 0.6 g C m^−2^ d^−1^ when upscaling to lakes (*n* = 361,777) located in permafrost-free area of Western Siberia (using same approach as described above for permafrost-affected lakes). We also estimated C yield using the same approach as above. We quantified C emission for ponds using the published median C emission rate for permafrost-affected (1.12 g C m^−2^ d^−1^) and permafrost-free (0.6 g C m^−2^ d^−1^) smallest size class lakes of Western Siberia, and multiplied this with extrapolated pond area and median season length for respective region. Since it has been recently suggested that pond area distribution in the landscape does not follow Pareto law^31,38^, we also quantified C emission for ponds using the fraction of land covered by these water bodies types for each of the permafrost zone derived from dataset available in Muster et al.^31^. Following this approach the total C emission for ponds is ~3.4-fold lower.

### Uncertainty in C emission estimates

The uncertainty in C emission rates values for the Ob’ main channel was estimated using a Monte Carlo approach. We randomly subsampled ten times 1000 values of each of the following variables: *p*CO_2 water_, *p*CO_2 atmosphere_, *k* and chemical enhancement factor, and estimated C emission rates as above. Using this approach, the mean C emission rate for Ob’ main channel is ~1.8-fold greater compared to the mean quantified C emission rate, whereas the median values are almost identical (Supplementary Fig. 4). To assess uncertainty in C emission from different components of inland waters in a uniform way, we used the standard rules of error propagation. We assumed 15% uncertainty in estimates of key variables (C emission rates, water areas, and season length) and propagated our error following:

2 $$\delta R = \left| R \right| + \sqrt {\left( {\frac{{\delta x}}{x}} \right)^2 + \left( {\frac{{\delta y}}{y}} \right)^2 + \left( {\frac{{\delta z}}{z}} \right)^2}$$

where *δR* is the uncertainty, *R* is a result of multiplication of C emission rates, water areas, and season lengths, while *δx*, *δy*, and *δz* are 15% uncertainty estimates of C emission rates (*x*), water areas (*y*), and season lengths (*z*), respectively. We then estimated the uncertainties for total river and lake C emission as well as total C emission from inland waters (rivers, streams, lakes, ponds) as follows:

3 $$\delta R = \sqrt {\left( {\delta x} \right)^2 + \left( {\delta y} \right)^2 + \left( {\delta z} \right)^2 + \ldots }$$

where *δR* is the total uncertainty, while *δx*, *δy*, and *δz*, … are C emission uncertainties estimated for each of the inland water components from Eq. (2).

### Statistics

We examined differences in inland water C emission and C yield (rivers + lakes, excluding streams and ponds) between different permafrost zones of Western Siberia with Kruskal–Wallis test on randomly subsampled 500 values. We considered the result statistically significant at *p* < 0.05.

### Net ecosystem exchange data

We used NASA SMAP L4 Global Daily 9 km EASE-Grid Carbon Net Ecosystem Exchange, Version 4 product^39^ (https://nsidc.org/data/SPL4CMDL) to quantify monthly and annual rates of NEE across Western Siberia. This product provides global gridded (9 × 9 km) daily estimates of NEE (CO_2_) derived using satellite data based on terrestrial C flux model informed by Soil Moisture Active Passive (SMAP) L-band microwave observations, land cover and vegetation inputs from the Moderate Resolution Imaging Spectroradiometer, Visible Infrared Imaging Radiometer Suite, and the Goddard Earth Observing System Model, Version 5 land model assimilation system. Note that NEE detects only terrestrial vegetation and ignores aquatic surfaces. We downloaded the data files covering the full year of 2016. Note that our sampling campaign on lakes was conducted during open water period (May–October) 2016 and the Ob River was studied in July–August 2016. We imported these data files to R, extracted the mean NEE rate as well as 1 s.d. of the mean NEE rate data from these data files (representing in total 730 individual data of h5 format), projected them, converted them to GeoTiff format, and clipped the area matching the location of Ob’, Pur, and Taz River basins (Supplementary Fig. 1). We then quantified the mean NEE rate and mean s.d. across all 71,280 of 9 × 9 km cells covering the region, for each day separately, and after that we estimated the NEE for entire Western Siberia as a sum of products of each 71,280 individual cells’ NEE rates and respective cells’ resolution (also for each day separately). The monthly NEE and annual NEE were quantified as the sum of daily and monthly NEE, respectively (Supplementary Table 2). We assessed uncertainty in monthly and annual NEE using Eqs. (2) and (3).

### Net C uptake by the Kara Sea

To estimate net C uptake by the Kara Sea, we used published grid (1 × 1 degree) data for CO_2_ uptake by the Arctic Ocean (60–90°N, 0°–360°) for year 2014^43,44^ (http://www.jamstec.go.jp/res/ress/yasunaka/co2flux/#!prettyPhoto). We downloaded the data file, imported it to R, and clipped the grid cells matching the GPS boundaries of the Kara Sea (2784 observations). We then quantified the mean annual CO_2_ uptake rate encoded in these grid cells (–7.64 mmol m^−2^ d^−1^, from January to December) and multiplied it by the water surface area of the Kara Sea and by 365 days (as done in Yasunaka et al.^44^).

## Supplementary information

Supplementary Information

## Data Availability

The datasets generated during this study are available in the ZENODO repository: 10.5281/zenodo.4153049.

## Code availability

Code for data analysis (for terrestrial NEE) is available upon request.
