# Supplementary material for: Carbon emission from Western Siberian inland waters
Source: Nat Commun. 2021 Feb 5;12:825. doi: 10.1038/s41467-021-21054-1 (PMC7864975; doi:10.1038/s41467-021-21054-1)
Supplement: Supplementary file 1 — Supplementary Information [file 41467_2021_21054_MOESM1_ESM.pdf]

Supplementary Information:

Carbon emission from Western Siberian inland waters

Jan Karlsson[1]*, Svetlana Serikova[1,2], Sergey N. Vorobyev[3], Gerard Rocher-Ros[1], Blaize Denfeld[1,4], Oleg S. Pokrovsky[3,5,6]

[1]Climate Impacts Research Centre (CIRC), Department of Ecology and Environmental Science, Umeå University, Linnaeus väg 6, 901 87 Umeå, Sweden.

[2]Swedish Geotechnical Institute, Olaus Magnus väg 35, 581 93 Linköping, Sweden.

[3]BIO-GEO-CLIM Laboratory, Tomsk State University, Lenina 36, 634 050 Tomsk, Russia.

[4]Swedish University of Agricultural Sciences, Uppsala, Lennart Hjelms väg 9, 75651 Uppsala, Sweden

[5]GET UMR 5563 CNRS, Geoscience and Environment, University of Toulouse, 14 Avenue Edouard Belin, 31400 Toulouse, France.

[6]Institute of Ecological Problems of the North, N. Laverov Federal Center for Integrated Arctic Research, Russian Academy of Sciences, Nab. Severnoi Dviny 23, 163 000 Arkhangelsk, Russia.

*Correspondence to: J. Karlsson (jan.p.karlsson@umu.se).

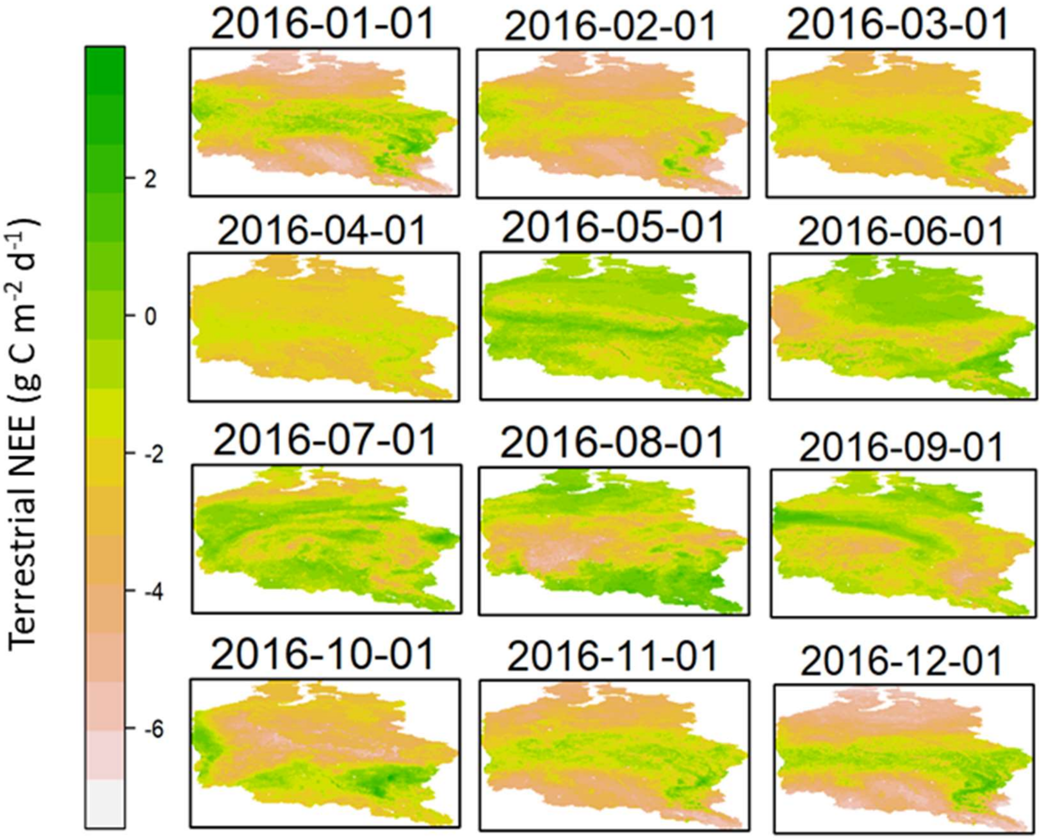

Supplementary Figure 1. Daily net ecosystem exchange (NEE) rate across Western Siberia for the first day of each month in 2016. Data for all days can be found in the data repository.

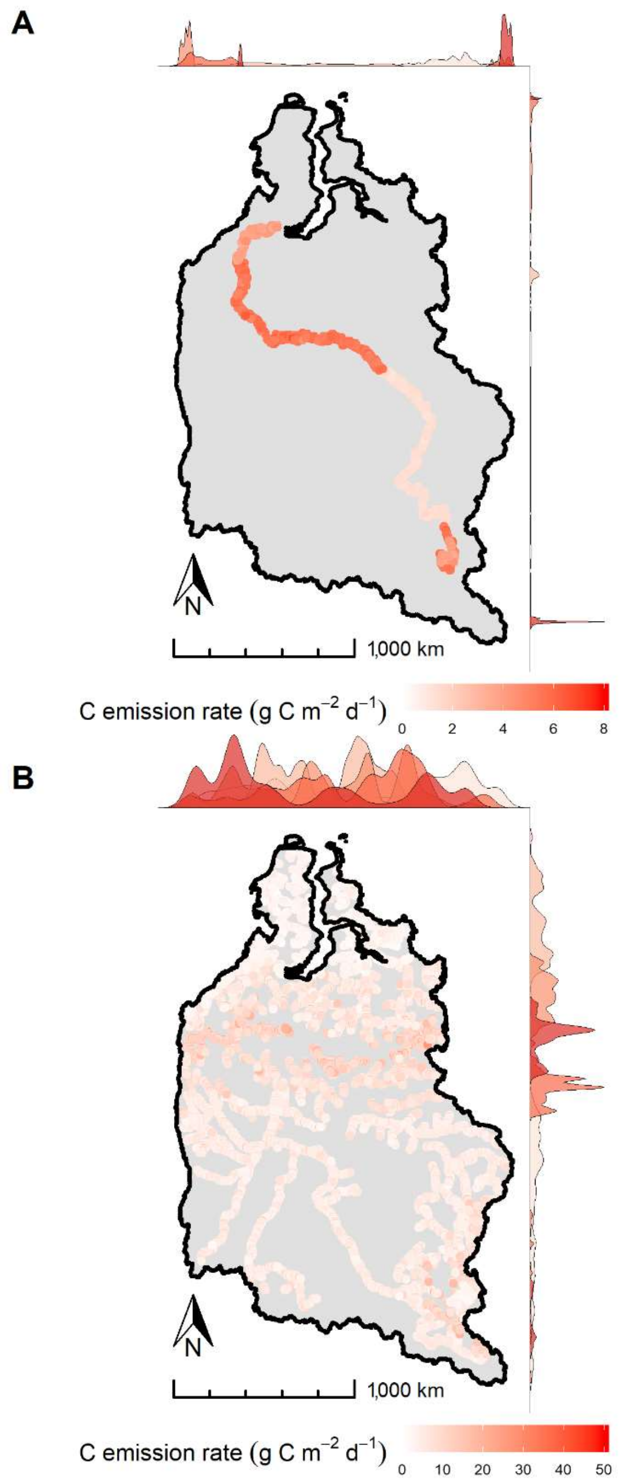

Supplementary Figure 2. Quantified C emission rates for the Ob' main channel (**A**) and for river network (**B**). The side panels represent color-coded density plots of the respective C emission rates across latitudes (vertical dimension) and longitudes (horizontal dimension).

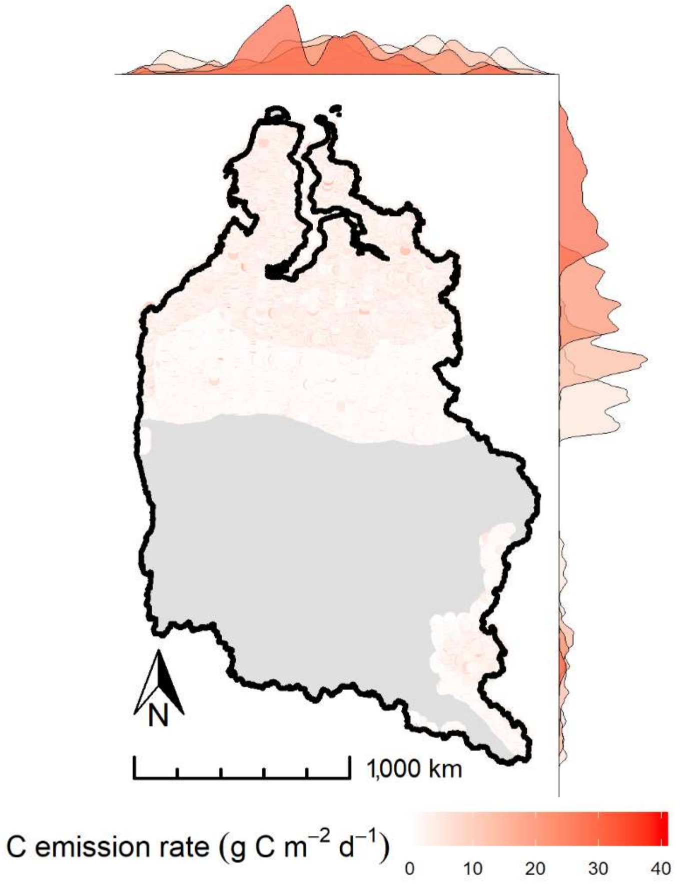

Supplementary Figure 3. Quantified C emission rates for permafrost-affected lakes. The side panels represent color-coded density plots of the respective C emission rates across latitudes (vertical dimension) and longitudes (horizontal dimension). Note that we removed 1 outlier to visually improve the graph. The data on lakes in the permafrost-free zone is not shown on the figure since it has been derived from Sabrekov et al. 2017[1].

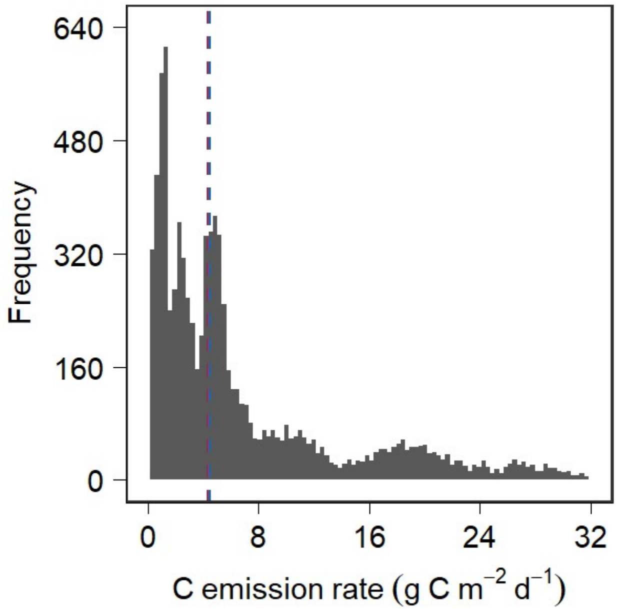

Supplementary Figure 4. Frequency distribution of estimated C emission rate for the Ob' main channel using Monte Carlo approach. The red line indicates the median of quantified C emission rate, whereas the blue line indicates the median of estimated C emission rate using Monte Carlo simulation.

Supplementary Table 1. Parameters of mean ± s.d. of different variables across permafrost zones of Western Siberia. Dash stands for not applicable, since the data for permafrost-free lakes were derived from Sabrekov et al. 2017[1].

	Permafrost zone				
	**Permafrost-free**	**Isolated**	**Sporadic**	**Discontinuous**	**Continuous**
**Ob' main channel**					
CO_2 emission rate (g C $m^{-2\,(water)}$ d^{-1})	1.31 ± 0.89	5.39 ± 0.67	5.41 ± 0.69	3.82 ± 1.14	3.82 ± 1.13
C yield (g C $m^{-2\,(land)}$ yr^{-1})	0.02	0.59	0.11	0.15	0.008
**Rivers > 90 m wide**					
CO_2 emission rate (g C $m^{-2\,(water)}$ d^{-1})	4.59 ± 3.70	7.45 ± 5.70	11.06 ± 14.39	5.88 ± 7.69	2.58 ± 1.72
C yield (g C $m^{-2\,(land)}$ yr^{-1})	3.52	2.78	6.57	4.89	2.26
**Lakes > 0.01 km² area**					
C emission rate (g C $m^{-2\,(water)}$ d^{-1})	-	1.11 ± 1.36	0.77 ± 1.38	2.76 ± 3.67	2.83 ± 3.13
C yield (g C $m^{-2\,(land)}$ yr^{-1})	3.79	8.95	7.58	34.2	20.4
Land area (km²)	2,278,980	343,473	360,404	357,125	303,307

Supplementary Table 2. Monthly net ecosystem exchange (NEE) of Western Siberia. The mean and s.d. represent a mean and 1 s.d. of NEE rate aggregated per month across entire Western Siberia (across 71,280 of 9 x 9 km cells covering the region), whereas the monthly NEE values are reported as a sum of products' sum of each 71,280 individual cells' NEE rates and respective cells' resolution. The NEE data was extracted from NASA SMAP L4 Global Daily 9 km EASE-Grid Carbon Net Ecosystem Exchange, Version 4 product (https://nsidc.org/data/SPL4CMDL)[2].

**Month**	**NEE**		
	Mean (g C $m^{-2\ (land)}\ d^{-1}$)	s.d. (g C $m^{-2\ (land)}\ d^{-1}$)	Total (Tg C $month^{-1}$)
January	0.54	0.001	61.56
February	0.59	0.006	62.84
March	0.64	0.016	73.26
April	0.61	0.056	67.72
May	-0.17	0.077	-20.17
June	-1.28	0.087	-140.60
July	-1.88	0.112	-213.38
August	-0.74	0.123	-198.18
September	-0.23	0.049	-26.06
October	0.26	0.023	30.32
November	0.44	0.004	48.77
December	0.48	0.002	55.30

Supplementary Table 3. Annual flow-weighted DOC and DIC export by the Ob', Pur and Taz river basins. DOC flux for Ob' is based on Kaiser et al. 2017[3] while DIC flux for Ob' is derived from Tank et al. 2012 (mean for the period of 2003-2009). DOC fluxes for Pur and Taz are based on Pokrovsky et al. 2015[4,5] (mean over the period of 2013-2014), whereas DIC fluxes for these rivers are derived from Gordeev et al. 1996[6] (both are quantified based on discharge data from 1971-1980).

**River**	**DOC flux**	**DIC flux**	**C flux**
	Tg C yr^{-1}	Tg C yr^{-1}	Pg C yr^{-1}
Ob'	3.91	5.9	0.0098
Pur	0.23	0.17	0.0004
Taz	0.28	0.58	0.0008

Supplementary Table 4. Parameters of linear regression between ice-free season length and latitude.

	**Dependent variable**	
	**Ice-free season length, days**	
**Predictor variable**	**(1)**	**(2)**
River latitude, °N	-6.53*** (0.07)	
Lake latitude, °N		-6.31*** (0.08)
Intercept	578.71*** (4.63)	536.47*** (5.27)
Observations	116	228
R^2	0.99	0.96
Adjusted R^2	0.99	0.96
Residual Std. Error	2.22 (df = 114)	2.36 (df = 226)
F Statistics	7,899.51*** (df = 1;114)	6,012.09*** (df = 1;226)
Note:		***p<0.01

References

1 Sabrekov, A. F. *et al.* Variability in methane emissions from West Siberia's shallow boreal lakes on a regional scale and its environmental controls. *Biogeosciences* **14**, 3715-3742, doi:10.5194/bg-14-3715-2017 (2017).

2 Kimball, J. S., Jones, L. A., Kundig, T. & Reichle, R. SMAP L4 Global Daily 9 km EASE-Grid Carbon Net Ecosystem Exchange, Version 4. doi:https://doi.org/10.5067/9831N0JGVAF6 (2018).

3 Kaiser, K., Canedo-Oropeza, M., McMahon, R. & Amon, R. M. W. Origins and transformations of dissolved organic matter in large Arctic rivers. *Scientific Reports* **7**, doi:10.1038/s41598-017-12729-1 (2017).

4 Pokrovsky, O. S. *et al.* Permafrost coverage, watershed area and season control of dissolved carbon and major elements in western Siberian rivers. *Biogeosciences* **12**, 6301-6320, doi:10.5194/bg-12-6301-2015 (2015).

5 Tank, S. E. *et al.* A land-to-ocean perspective on the magnitude, source and implication of DIC flux from major Arctic rivers to the Arctic Ocean. *Global Biogeochemical Cycles* **26**, doi:10.1029/2011gb004192 (2012).

6 Gordeev, V. V., Martin, J. M., Sidorov, I. S. & Sidorova, M. V. A reassessment of the Eurasian river input of water, sediment, major elements, and nutrients to the Arctic Ocean. *American Journal of Science* **296**, 664-691, doi:10.2475/ajs.296.6.664 (1996).
